# Chiromancy

**Published:** 1885-05

**Authors:** 


### Chiromancy.

Most people are aware that chiromancy has been practiced from the remotest times, and appears to have come from China through India and Egypt into Europe at some unknown period. During the present century this occult science has been taken up and formulated until it is said to have become more or less practicable to people who are neitherwizards nor impostors. The fact is, some are endeavoring to reduce it from an occult to an exact science, and this endeavor is the cause of the fallacy of modern books on the subject. It is not an exact science any more than art is science. By the minute and careful observation of an artist's eye, and by the assistance of the accurate and ready memory of a judge's brain, enough data may be amassed whereby a man with the power of creation of a poet can "tell a hand" and set forth the character in well chosen words after a few minutes' study of the palms and fingers. Yet the definite rules employed are no more definite than those belonging to any other art. One set of lines for chiromancy runs as follows: Open the left hand—half way between the thumb and forefinger there begins, on the very edge of the palm, a line which runs in a quarter circle to the middle of the wrist. This is the famous line of life. If it be unbroken and clearly marked, it indicates long life and health. From the same point joining it another line runs through the middle of the palm. This is the line of the head or of intellect. Almost parallel with it there is a third line beginning on the other side below the little finger, and leading, when well developed, to the root of the forefinger. This is the line of the heart. If clear, deep and even it indicates a good capacity for honorable love and warm affections. It is most favorable when this line, as well as that of the head, has a fork or branch at the end. From the line of life at the wrist there ascends a fourth line, known at that of fate, saturn or fortune. When it rises as far as the middle finger it is said to promise excessive good luck or prosperity. From the same point at the wrist there goes toward the middle finger a line called by some the hepatic or liver line and by others the *Via lactea,*

also the *Via lasciva.* There is great confusion even in Desbarolles himself, as to these lines, whether there are two of them, and which is which. When a large triangle is formed by the lines of life, the head and the liver, with one èven right angle and two acute angles, it indicates breadth and energy of character. If this triangle be divided into two by the line of fate, the subject will be susceptible of high intellectual culture; if the lesser triangle contain one or two more, there will be genius, and a capacity for knowledge. If the first joint of the thumb be long and round, and the nail in it cushioned in the flesh, it denotes obstinacy, but if the next joint be also very long, reason and reflection will convert the obstinacy to a creditable firmness. A line from the ring finger downward is a sign of a gift for art in one or all branches. Lines on the wrist are called the bracelet of Venus, and are said by some chiromancers to indicate each 30 years of life. All lines correct and balance one another.—*The Saturday Review.*

LACTOPEPTINE has been so generally tested, and has become so well established as a digestive of the highest power, that practitioners ought not to be without it. Indeed, it is of more frequent service in digestive troubles than any othêr one remedy which has been brought to professional attention. Its ormula, as published upon every package, commends itself; and the experience of the most eminent practitioners in America and England especially attest its great utility. In several cases in which its use was thought to be indicated, we have been surprised at the marked benefit following its administration. *Lactopeptine* already contains hydrochloric and lactic acids—the other important physiological digestives—pancreatine, diastase and sugar of milk.

